# The Association of Light Physical Activity With Health Indicators Among Middle-Aged and Older Adults

**DOI:** 10.1093/geroni/igaa057.616

**Published:** 2020-12-16

**Authors:** Kristen Moore, Kaigang Li, Manfred Diehl, Katherine Thompson, Abigail Nehrkorn-Bailey

**Affiliations:** 1 Colorado State University, Fort Collins, Colorado, United States; 2 University of Chicago Medicine, Chicago, Illinois, United States

## Abstract

Physical Activity (PA) Guidelines for Americans recommend engagement in moderate or moderate-to-vigorous physical activity (MVPA) for middle-aged and older adults. Although these guidelines encourage adults to “move more and sit less,” there are no explicit recommendations for engaging in Light PA (LPA). The purpose of this study was to examine the association between LPA and health Indicators among middle-aged and older adults, in the context of sedentary time (ST) and MVPA. We used baseline data from 171 individuals (Mean age: 59.3±8.51 years), participating in Colorado State University’s AgingPlus program. Selected health Indicators included Body Mass Index (BMI), waist circumference (WC in cm), mean arterial pressure (MAP in mmHg), grip strength (GS in lbs), and indirect VO2max (ml/kg/min). ST and PA were measured using Actigraph accelerometers worn for 7 days (excluding wear time <500 mins/day). Linear regression analyses, controlling for sex, age group, and race, indicated that more ST was associated with greater BMI (B=3.33), greater WC (B=3.35), and lower VO2max (B=-3.09). More LPA was associated with lower BMI (B=-4.47), lower WC (B=-5.0), lower MAP (B=-2.81), and higher VO2max (B=4.64). MVPA was associated with lower BMI (B=-3.04), lower WC (B=-4.21), higher VO2max (B=4.74), and higher grip strength (B=2.33). In conclusion, more ST was associated with indicators of poorer physical health. LPA, similar to MVPA, was associated with indicators of better physical health and performance. Future longitudinal and experimental studies examining the causal relationship between LPA and physical health and performance are warranted.

